# Supplementary material for: Assessment of content validity and psychometric properties of VISA-A for Achilles tendinopathy
Source: PLoS One. 2021 Mar 11;16(3):e0247152. doi: 10.1371/journal.pone.0247152 (PMC7951845; doi:10.1371/journal.pone.0247152)
Supplement: Attachment — Submitted filename: response to reviewer jan 6 2021.docx [file pone.0247152.s002.docx]

We thank the reviewer for these very relevant comments and we address them in the following:

1. (Line 119-120) Can the author provide citation or explain the reason(s) of having such cut-off point for age, BMI and duration of symptoms?

Response: The following has been added to the text at line 121 on page 5: “For DIF analyses, the cutoff of +/- 44 years of age was chosen because the median age for the sample group was 43.6 years. This allowed for a dichotomization of younger versus older persons for comparison of scoring patterns across the groups. For BMI, the value of 25 was chosen, as this was also the median value for the group. The duration of symptoms grouping was chosen to allow for a comparison of scoring patterns across groups that might be expected to have different levels of severity of symptoms (e.g., less than 3 months would be acute symptoms, 4 to 12 months would approach chronic symptoms, and more than 12 months would be manifest chronic tendinopathy).”

2. (Table 4) Would suggest to standardize the decimal places for the numbers. In addition, suggest to keep to one p-value, unless there's multiple category (e.g. in the sex category, the same p-value appeared twice)

Response: The bold values, which were the statistically significant values, have now been standardized. We hope that this addresses the concerns of the reviewer.

3. (Line 251) Suggest to standardize the numbers in one sentence (i.e. eight, three and 3)

Response: the words three and eight have been changed to 3 and 8. Thank you for pointing this out.

4. Based on my understanding, VISA-A questionnaire measures clinical severity of Achilles tendinopathy. Throughout the paper, it was not clearly mentioned about what VISA-A measures (mentioned in the paper "VISA-A measures Achilles tendinopathy, which is slightly confusing). Perhaps, the authors could clearly explain it in the introduction and conclusion, so that the readers are clearer.

Response: The creators of VISA intended for it to measure tendinopathy but we have shown that it really does not do that, so we do not have a good response to this enquiry.
